# Elucidating the immunomodulatory roles and mechanisms of CUL4B in the immune system: a comprehensive review

**DOI:** 10.3389/fimmu.2025.1473817

**Published:** 2025-03-31

**Authors:** Siyu He, Ziyue Wang, Yinan Zhu, Mingfang Sun, Xuyong Lin

**Affiliations:** Department of Pathology, The First Hospital of China Medical University, Shenyang, Liaoning, China

**Keywords:** CUL4B, immune system, CRL4B, cancer immunology, epigenetic regulation

## Abstract

Cullin 4B (CUL4B), a pivotal member of the Cullins protein family, plays a crucial role in immune regulation and has garnered significant research attention. CUL4B, through the Cullin 4B-RING E3 ubiquitin ligase (CRL4B) complex, regulates CD4+ T cell differentiation, fostering a balance between TH1 and TH2 subsets, and expedites DNA damage repair to bolster T cell persistence. In B cells, CUL4B upregulation stimulates immune responses but is linked to an unfavorable prognosis in lymphoma. In innate immunity, CUL4B modulates Toll-like receptor (TLR)-mediated anti-inflammatory responses, enhancing macrophage migration and adhesion. CUL4B also plays a role in potentiating anti-tumor immunity by restricting the activity of myeloid-derived suppressor cells (MDSCs). In disease pathogenesis, CUL4B limits MDSCs to enhance anti-tumor effects, and its inhibition in experimental autoimmune encephalomyelitis (EAE) models have demonstrated beneficial effects, underscoring its potential therapeutic significance in autoimmune diseases. Furthermore, CUL4B is involved in various immune-related cancers and inflammation, including pleural mesothelioma, human osteosarcoma, and colitis-associated cancer. In metabolic diseases, CUL4B regulates adipose tissue and insulin sensitivity, with its depletion improving metabolic phenotypes. This review highlights the pivotal role of CUL4B in maintaining immune homeostasis and provides novel perspectives and insights into the understanding and development of treatments for immune-related disorders.

## 1 Introduction

**Figure 1:**
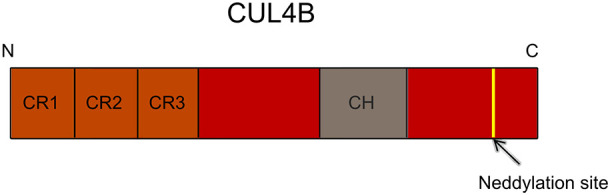
Schematic diagram of CUL4B domain architecture. The N-terminus of CUL4B encompasses three critical cullin repeats (CR1-CR3), pivotal for its specific functions. The C-terminus highlights a key Neddylation site, central to CUL4B’s Neddylation, regulating its activity and function via Nedd8 binding. CR, cullin repeat; CH, cullin homology domain.

Cullin 4B (CUL4B) possesses a distinctive cullin homology domain (CH), a highly conserved region of approximately 200 amino acids. Its amino-terminal domain (NTD) exhibits an elongated, stalk-like structure, comprised of three reiterated sequences known as cullin repeats (CR1 to CR3). Conversely, the carboxyl-terminal domain (CTD) adopts a globular conformation, surrounding which lies a Neddylation site. The site facilitates a pivotal covalent attachment, involving the ubiquitin-like protein Nedd8, which subsequently triggers profound conformational transformations within CUL4B(**Figure 1**) (1, 2).

**Figure 2:**
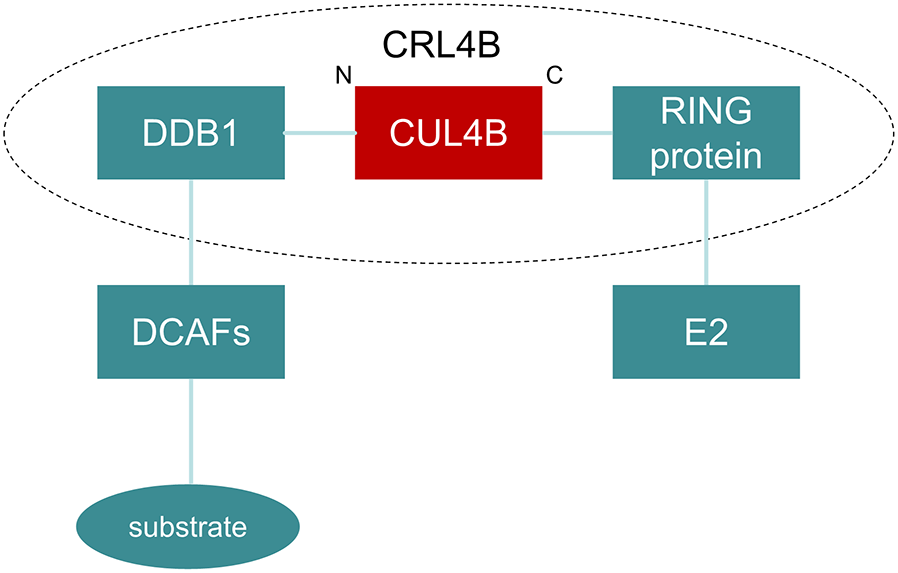
Structure of CRL4B. In the CRL4 complex, CUL4B is connected to the RING protein (Rbx1/ROC1) through its carboxyl-terminal end, which is responsible for recruiting E2 ubiquitin-conjugating enzymes. On the other end, the amino-terminal of CUL4B binds to DDB1, which utilizes DCAFs to recognize specific substrates, thereby facilitating their ubiquitination process.

The unique structural features of CUL4B enable its assembly into the Cullin 4B-RING E3 ubiquitin ligase (CRL4B) complex. This complex, with CUL4B serving as the central scaffold, intricately interacts with a diverse array of components, including the RING protein (Rbx1/ROC1), Damage-specific DNA-binding protein 1 (DDB1), and various DDB1- and CUL4-associated factors (DCAFs) (**Figure 2**). Research has disclosed that the CRL4B complex assumes a pivotal role in a diverse range of biological and pathological processes, including neural development, adipogenesis, and tumorigenesis and progression, primarily through two distinct mechanisms. Firstly, it facilitates the multi-ubiquitin tagging of substrate proteins, thereby triggering their subsequent degradation (2). Secondly, it catalyzes the monoubiquitination of H2A at the K119 site, which elicits the engagement of Polycomb Repressive Complex 2 (PRC2) (3).

While both CUL4B and Cullin 4A (CUL4A) share structural similarities and participate in ubiquitin ligase complexes, CUL4B has garnered increasing attention for its unique roles in immune regulation. Specifically, CUL4B’s involvement in T cell differentiation, B cell function, and innate immune responses has been highlighted in recent studies (4–6). In contrast, CUL4A is more extensively studied in DNA damage repair and cell cycle regulation (7–9). This review aims to provide a comprehensive overview of the current understanding of CUL4B’s roles in immunity (**Table 1**), contrasting it with the well-characterized functions of CUL4A. By exploring the latest findings and discussing potential avenues for future research, we hope to contribute to the ongoing efforts to unravel the complex mechanisms underlying immune regulation and disease pathogenesis.

**Table 1:** Cellular roles of CUL4B in different pathological conditions.

Pathological Condition	Cell Type	Role of CUL4B
Adaptive Immunity	CD4+ T cells	Promotes DNA damage repair, regulates TH1 and TH2 cell differentiation, and maintains T cell persistence.
	CD8+ T cells	Regulates DNA damage response and cell cycle progression, enhancing antiviral immune responses.
	B cells	Upregulation of CUL4B enhances immune responses in germinal center B cells, promoting γ-herpesvirus infection and lymphoma cell proliferation.
Innate Immunity	Macrophages	Suppresses TLR-mediated inflammatory responses, promotes macrophage migration and adhesion, and regulates the production of anti-inflammatory cytokine IL-10.
Tumor Immunity	Myeloid-derived suppressor cells (MDSCs)	Inhibits the accumulation and activation of MDSCs, reduces immunosuppression in the tumor microenvironment, and enhances anti-tumor immune responses.
Metabolic Diseases	Adipocytes	Negatively regulates PPARγ, promotes adipogenesis and insulin sensitivity, and improves metabolic phenotypes.
Autoimmune Diseases	T cells	Regulates CD4+ T cell expansion and differentiation, and is involved in the pathology of autoimmune diseases such as rheumatoid arthritis.
Inflammation-Related Cancers	Pleural mesothelioma cells	Overexpression of CUL4B is associated with poor prognosis, while downregulation reduces tumor cell proliferation and increases cell death.
	Human osteosarcoma cells	Enhances CRL4BDCAF11 E3 ligase activity through the TNF-α/NF-κB axis, regulating cell cycle progression.
	Colitis-associated cancer (CAC) cells	Inflammation and DNA methylation-dependent downregulation of miR-34b-5p mediates c-MYC expression and CRL4DCAF4 E3 ligase activity, promoting tumor development.
Experimental Autoimmune Encephalomyelitis (EAE)	T cells	CUL4B suppresses autoimmune responses in EAE models, indicating potential therapeutic significance.

Chart Explanation:

Adaptive Immunity: CUL4B plays a crucial role in T and B cells, regulating immune responses and cell differentiation.

Innate Immunity: CUL4B modulates macrophage function to suppress excessive inflammatory responses.

Tumor Immunity: CUL4B enhances anti-tumor immunity by inhibiting the accumulation and activation of MDSCs.

Metabolic Diseases: CUL4B regulates metabolic balance in adipocytes, improving insulin sensitivity.

Autoimmune Diseases: CUL4B is involved in T cell regulation, influencing the pathology of autoimmune diseases.

Inflammation-Related Cancers: CUL4B promotes or inhibits tumor development through various mechanisms in different cancers.

Experimental Autoimmune Encephalomyelitis (EAE): CUL4B shows potential in suppressing autoimmune responses in EAE models.

## 2 The role of CUL4B in adaptive immunity

### 2.1 The functionality of T-cells in immune response

The core mechanism of adaptive immunity lies in the clonal proliferation of pathogen-specific T-lymphocytes (10, 11). For T-cells to generate a sufficient quantity to combat viral and certain bacterial infections, they must proliferate at an expedited rate, necessitating hyperkinetic cell division (12, 13), coupled with expedited DNA replication and a markedly abbreviated cell cycle duration. Proliferative cells, and particularly T-cells, must possess sophisticated mechanisms to detect and promptly repair DNA lesions that occur during replication. Without efficient DNA repair mechanisms in place, activated T-cells are greatly vulnerable to replication-induced genomic instability and deleterious DNA alterations (14, 15). Unrepaired DNA damage can disrupt the normal cell cycle progression, triggering programmed cell death, which not only weakens the body’s ability to combat invading pathogens but also ultimately compromises the host’s immunological defense mechanisms.

### 2.2 CUL4B regulates CD4^+^ T cell function and differentiation

CD4+ T cells are also known as Th cells or T assistant cells, which play a role in assisting other immune cells during immune responses. Upon encountering antigenic stimulation, naïve CD4+ T cells embark on a transformational journey, expanding in size and initiating rapid proliferation as they enter the cell cycle. This proliferative burst is followed by a differentiation process, where the cells meticulously adapt to the diverse cytokine microenvironments. Within these milieus, they give rise to distinct effector CD4+ T cell subsets, each tailored to specific immune responses. These include effector helper T cells, like TH1, TH2, TH17, and TFH, whose development is intricately tied to the surrounding cytokine landscape. Additionally, a subset of these cells differentiates into regulatory T cells (Treg), tasked with modulating the immune response to maintain homeostasis and prevent excessive inflammation (16–18). Upon antigen decline, effector CD4+ T cells predominantly undergo apoptosis, a widely reported phenomenon (19). Survivors transition into memory CD4+ T cells, which are crucial for immunological memory establishment and future immune preparedness (20). Complex checkpoint mechanisms tightly control this transition, ensuring immune response cessation and memory maintenance. Notably, the G2/M checkpoint halts DNA-damaged cells from mitosis, preserving genetic integrity and division accuracy (21). Unfixed DNA harm builds up, prompting cell cycle halt or programmed death (14).

Ensuring the non-propagation of flawed DNA, facilitating the process of DNA repair, and maintaining the stability of the genetic blueprint are key functions upheld by the damage-specific DNA-binding protein 1 (DDB1), which occupies a pivotal role in these processes (7–9). DDB1, alongside DDB2, this heterodimer, referred to as UVDDB, plays a pivotal role in identifying DNA damage that is elicited by ultraviolet radiation, within the intricate mechanism of nucleotide excision repair(NER), commonly referred to as the NER pathway (22). DDB1 performs a crucial function as a fundamental element within the CRL4 complex, which encompasses the Cul4A-RING ubiquitin E3 ligase. It operates as an adaptor molecule, linking Cullin 4A (Cul4A) seamlessly to the array of DCAFs, thereby facilitating the intricate interactions essential for the complex’s function (5). Here, we primarily discuss Cul4A-RING ubiquitin E3 ligase because the current literature provides more extensive data on Cul4A, whereas research on CUL4B in this specific context is still emerging. Experimental results emphasize the role of DDB1 in enhancing the proliferation of T lymphocytes by preventing the interruption of the cell cycle and the induction of cell demise resulting from DNA damage (4). The NER pathway effectively remedies DNA injury without hindering cellular cycle advancement. Nevertheless, alterations affecting the constituents of NER, particularly the absence of DDB1, lead to an irregular buildup of DNA harm. Subsequently, it elicits the activation of both the ATM/ATR-Chk1 and p53 signaling cascades, which collaboratively induce cellular cycle halt and programmed cell demise. In the absence of DDB1, stimulated CD4+ T lymphocytes undergo a cell cycle halt specifically during the G2-M transition phase, demonstrating heightened levels of cell death (4).

Similar phenotypic traits to those seen in DDB1-deficient mice are exhibited by mice with concurrent deletions of CUL4A and CUL4B genes within their population of activated CD4+ T cells (6). The pivotal role of DDB1 in genomic stability and the development of helper T cells is underscored by this observation, stemming from its participation as a constituent within the E3 ubiquitin ligase complex.

A thorough scientific examination has revealed the essential dependence of stimulated and multiplying CD4+ T lymphocytes on the E3 ubiquitin ligase CUL4B for the expedited remediation of DNA injuries (6).In the context of CD4+ T cell activation, CUL4B demonstrates a strong interaction with Vprbp, additionally designated as DCAF1, the DDB1-CUL4 Associated Factor 1. Initially recognized as the binding counterpart of the HIV-1 Vpr protein, VprBP (23), DCAF1 falls within the DCAF family and engages in a diverse range of physiological and pathological functions (24). These include, but are not limited to, cellular growth and proliferation (25), viral replication (26), and transcriptional repression (27). Remarkably, the intricate connections of both CUL4B and DCAF1 with key proteins, including MRE11A, RAD50, and SMC1A, serve as compelling evidence for their vital roles in the intricate machinery of DNA damage recognition and subsequent repair processes. The depletion of pSMC1A, the phosphorylated form of SMC1A, in mammalian cells has been conclusively demonstrated to elicit checkpoint deficiencies, compromised survival rates, and heightened chromosomal aberrations after DNA damage (28). While the absence of CUL4B does not alter the overall levels or chromatin localization of SMC1A, it significantly disrupts the process of ubiquitinating and phosphorylating SMC1A. In T lymphocytes of the CD4+ subtype lacking CUL4B, the phosphorylation status of Smc1a is diminished, which coincides with an increased expression of apoptotic genes, such as p21, Bbc3 (alternatively designated as Puma), and Bax.

Extensive experimental findings emphasize the synergetic function of CUL4B and DCAF1 in accelerating the remediation of DNA injuries that occur during duplication, ultimately fortifying the persistence and growth of CD4+ T cell colonies (6).

The secretion of interferon-gamma (IFN-γ), a pivotal regulator in mediating immune responses against intracellular pathogens and a contributing factor in autoimmune disease mechanisms, is induced by the progression of CD4+ T cells towards TH1 differentiation (29). On the other hand, cytokines, namely interleukin-5 (IL-5), interleukin-13 (IL-13), and interleukin-4 (IL-4), secreted by TH2 cells that differentiate from CD4+ T cells, are crucial in combating extracellular parasitic infections and orchestrating allergic reactions (30).In the specialized research conducted by Qin et al (31), it was firmly established that CUL4B functions as a pivotal transcriptional repressor, intricately modulating the precise choreography of lineage-specific transcription factors during the developmental progression of TH1 and TH2 cells into distinct lineages, and preserving their unique lineage identities after differentiation. Remarkably, the *in vitro* absence of CUL4B, as observed experimentally, highlighted its crucial function in preserving the stability of TH1 and TH2 cells, while simultaneously augmenting the plasticity between these distinct lineages. The study further revealed that the loss of CUL4B, both within *in vivo* and *in vitro* systems, elicited an enhanced differentiation of both TH1 and TH2 cells. This phenomenon was attributed to the association of CUL4B complexes with the promotor sequences of transcription factors specific to cell lineages within naive CD4+ T-lymphocytes, which simultaneously sustained heightened degrees of histone modifications, notably the monoubiquitination of H2AK119ub1 and the trimethylation of H3K27me3. During the commitment to a TH1 lineage, CUL4B dissociates from the Tbx21 promoter, accompanied by a decrease in H2AK119ub1 and H3K27me3 modifications, thereby permitting the transcription of Tbx21. Tbx21, also known as T-bet, is a key transcription factor that drives the differentiation of naive CD4+ T cells into TH1 cells and is essential for the production of IFN-γ (29). Analogously, during TH2 differentiation, CUL4B detaches from the Maf and TH2 cytokine promoters, with a corresponding reduction in H2AK119ub1 and H3K27me3 levels, enabling the transcription of TH2-associated factors. Maf is a transcription factor that, along with other factors, promotes the differentiation of naive CD4+ T cells into TH2 cells and is crucial for the production of cytokines such as IL-4, IL-5, and IL-13 (30).

At the Tbx21 and Maf loci, the CUL4B-RING E3 ligase complex and the PRC2 complex synergistically function, with the former promoting the monoubiquitination of H2AK119 and the latter catalyzing the trimethylation of H3K27, collectively modifying these sites epigenetically. This effectively represses their transcription during the differentiation of TH cells. This CRL4B-mediated placement of H2AK119ub1 functions as a vital protective mechanism to prevent the abnormal manifestation of genes specific to the TH1 and TH2 lineages, thereby safeguarding the accuracy and precision of the differentiation process (31).

### 2.3 CUL4B regulates CD8^+^ T cell function

The eradication of pathogens fundamentally hinges upon the cytokines dispatched by cytotoxic CD8+ T lymphocytes (CTLs), as well as the immediate termination of diseased cellular entities. Durable memory CD8+ T cell populations, on the other hand, confer enhanced protection against recurrent infections. The intricate transformation of naive CD8+ T lymphocytes into their memory counterparts, traversing the vital stages of immune response initiation, proliferation, and subsequent regression, is meticulously directed by an intricate interplay of cellular surface receptors, soluble signaling molecules, and sophisticated transcriptional machinery, all of which are intricately interconnected with profound metabolic adjustments (32).

Experimental findings underscore the critical function of CUL4B in modulating the DNA damage response (DDR) and cell cycle progression in CD8+ T cells, thereby facilitating their antiviral immune function (33). Specifically, the absence of CUL4B triggers a significant hindrance in the differentiation of CD8+ T cells into effector and memory cells immediately following viral infection, a phenomenon intimately associated with a heightened level of DNA damage. C-Myc, a transcription factor renowned for its pro-proliferative properties, exerts its influence by regulating the expression of numerous cellular pathways essential for S-phase progression. It achieves a delicate balance between replication stress and the activation of DDR (34–37). Following T cell receptor (TCR) activation, c-Myc upregulates CUL4B expression, which subsequently modulates the levels of p21 (Cdkn1a, a CDK inhibitor and cell cycle regulator) and Cyclin E2. Notably, p21 is subject to ubiquitination and degradation (38), and CUL4B promotes this degradation in CD8+ T cells.

The deficiency of CUL4B in CD8+ T cells also leads to increased levels of p21, a protein that functions as a CDK inhibitor, and the transcription factor Cyclin E2, which creates genomic instability. P21 does not have enzymatic activity but acts by inhibiting the activity of cyclin-dependent kinases (CDKs), thereby regulating the cell cycle. This further underscores the critical role of CUL4B in preserving genomic stability and potentiating the immune responses of antiviral CD8+ T lymphocytes, with c-Myc functioning as a key modulator of CUL4B expression in this regard.

### 2.4 CUL4B orchestrates B cell function

Within B cells, the upregulation of CUL4B boosts the immune reactions of germinal center(GC) B cells that are triggered by antigens (39).GC is a pivotal microenvironment in secondary lymphoid tissues, shaped by the interplay of B cells, antigens, and T cells, and holds great significance for humoral immune responses. Gammaherpesviruses, specifically Murine Gammaherpesvirus 68 (MHV68, also known as γHV68 or MuHV-4), Human Kaposi’s Sarcoma-associated Herpesvirus (KSHV), and Human Epstein-Barr Virus (EBV), infiltrate immature B cells, manipulate their signaling cascades, proliferate within the GC, thereby influencing the establishment of immune memory (40–42).

During the process of establishing latency, GC B cells emerge as the primary reservoir harboring latent viral genomes (43–45), hereby playing a fundamental role in the viral lifecycle and pathogenesis. In a specialized study examining the functional role of CUL4B in gammaherpesvirus infection, investigators employed CUL4B conditional knockout (CKO) murine models and subjected them to MHV68.H2bYFP viral challenge. The outcomes of this investigation highlighted profound alterations within the GC B cell (GC B cell; defined by the phenotypic markers B220+, GL7+, and CD95+) compartment of these mice. B cell-specific CUL4B KO mice had comparable numbers of GC B cells to WT mice under uninfected conditions. The number was only six-fold reduced under infected conditions (39). Under viral infection conditions, the virus-infected (virus-positive) GC B cells (identified as B220+, GL7+, CD95+, and YFP+) in CKO mice declined markedly, specifically by 116-fold compared to wild-type controls. The YFP+ indicates that these cells express yellow fluorescent protein (YFP), which is driven by a viral promoter, allowing for the visualization and quantification of infected cells (39). Additionally, the comprehensive frequency of GC B cells in CKO mice diminished by three-fold, whereas the prevalence of specifically infected GC B cells dropped by fifteen-fold. These alterations were accompanied by significantly diminished levels of antigen-specific antibodies, indicative of compromised humoral immune responses.

The expansion of GC B cells during gammaherpesvirus infection is critically dependent on the specific expression of CUL4B in B cells, as underscored by these collective findings, a process that is indispensable for the successful establishment of viral latency in peripheral tissues and the perpetuation of chronic gammaherpesvirus infections *in vivo*. However, the intricate molecular pathways mediating CUL4B’s facilitation of GC B cell infection or expansion remain elusive and necessitate further scrutiny to precisely determine their roles in the pathogenesis of gammaherpesvirus infections (39). Ying et al (46), conducted a study demonstrating that in the context of diffuse large B-cell lymphoma (DLBCL), elevated levels of CUL4B at both the tissue and cellular levels are a prominent feature. Mechanistically, CUL4B fosters DLBCL cell proliferation via the intricate choreography of autophagy, which is intimately tied to the initiation of signaling events orchestrated by JNK (c-Jun N-terminal kinase). This enhancement of growth is corroborated by clinical observations, where patients exhibiting heightened CUL4B expression display significantly shorter overall survival (OS) intervals compared to those with lower CUL4B levels.

Furthermore, the targeted downregulation of CUL4B through gene silencing strategies was found to effectively curb DLBCL cell proliferation, arrest the cell cycle, and attenuate cellular motility *in vitro*. More importantly, the abrogation of CUL4B function significantly hindered tumor progression in a DLBCL xenograft mouse model, underscoring its potential as a crucial therapeutic target. These discoveries, taken as a whole, emphasize the crucial function of CUL4B as a potential indicator and an innovative treatment approach for DLBCL management.

## 3 The role of CUL4B in innate immunity

The notion of inherent immunity denotes the primary line of host defense that comes into play within the initial hours of microbial encounter, serving as the first barrier to limit infection (47). The innate immune system is phylogenetically conserved and ubiquitous across virtually all multicellular organisms, playing a pivotal role in the detection of invading pathogens (48). Pattern recognition receptors (PRRs) on innate immune cells facilitate the recognition of pathogen-associated molecular patterns (PAMPs), enabling the cells to initiate an immune response. Following recognition, these cells release chemoattractants, inflammatory mediators, or antibacterial peptides to eradicate pathogens and summon additional immune cells to the targeted location (48, 49).

A pivotal process in innate immune recognition of pathogen invasion entails the engagement of TLRs, sensors that discern specific molecular signatures within microbial structures. Activation of these diverse TLRs triggers the orchestration of unique gene expression profiles, thereby igniting innate immune responses and directing the maturation of antigen-tailored adaptive immunity (50). The defining structural features of TLRs encompass an intracellular toll/interleukin-1 receptor (TIR) domain, along with an extracellular region rich in leucine repeats, known as the leucine-rich repeat (LRR) domain (48). Notably, TLR4 can discern the primary constituent of Gram-negative bacterial cell walls, namely lipopolysaccharide (LPS), triggering two signaling cascades: One involving myeloid differentiation primary response 88 (MyD88) and toll-interleukin 1 receptor domain-containing adaptor protein (TIRAP) on the plasma membrane, and another with TIR-domain-containing adapter-inducing interferon-β(TRIF) and TRIF-related adaptor molecule (TRAM) located within early endosomes following receptor endocytosis (51). During infection, TLR4 responds to LPS, eliciting a pro-inflammatory response to eradicate invading bacteria (52).TLR activation, albeit crucial for the defense mechanisms of the host, can elicit exaggerated immune and inflammatory reactions, ultimately resulting in damage to tissues and severe health complications akin to septic shock (53). Enhanced TLR signaling in macrophages heightens mouse susceptibility to endotoxin shock and sepsis caused by Escherichia coli (54). Additionally, increased IL-6 production predisposes mice to autoimmune diseases (55).

CUL4B serves as a suppressive modulator of TLR-triggered immune and inflammatory reactions. Its deficiency leads to distinct outcomes in different studies. On one hand, Song and colleagues demonstrated that CUL4B protects against excessive inflammatory reactions by inhibiting the synthesis of proinflammatory mediators while fostering the production of anti-inflammatory factors, thereby averting the overstimulation of TLR signaling pathways (53). Mechanistically, the inhibition of PTEN transcription by the CUL4B complex subsequently potentiates AKT activation, leading to decreased GSK3β activity. GSK3β, a crucial junction in signaling networks that orchestrate various cellular functions such as metabolic regulation, cell growth, cell fate determination, and developmental processes (56–58), plays a pivotal role in facilitating the generation of proinflammatory cytokines across multiple TLR signaling cascades (59). Pharmacological inhibition of GSK3β effectively blocks the enhanced inflammatory response triggered by TLR in CUL4B-deficient conditions, both *in vitro* and *in vivo*, and also mitigates TLR-induced mortality associated with CUL4B deficiency (53). The AKT-GSK3β-CREB(CAMP response element-binding protein) pathway serves as a key controller of IL-10 generation during TLR stimulation (60), CUL4B significantly facilitates CREB signaling in macrophages, a process that is pivotal for the production of the anti-inflammatory cytokine IL-10, while simultaneously restricting NF-κB signaling. Thus, CUL4B promotes the generation of anti-inflammatory cytokines, further underscoring its complex and multifaceted role in immune regulation (53).

On the other hand, prior paradoxical studies reported that bone marrow-specific ablation of CUL4B potentiates LPS-mediated acute peritonitis by augmenting the recruitment of key leukocyte populations, such as macrophages, neutrophils, and lymphocytes (61). However, this phenomenon is accompanied by an unexpected attenuation in the generation of proinflammatory cytokines TNF-α and IL-6, despite the enhanced DNA replication, cellular proliferation, and chemokine generation observed within macrophages. Pfeiffer et al. further elucidated this paradox by demonstrating that knocking down CUL4B through shRNA in RAW264.7 mouse macrophages upon LPS stimulation elicits a marked decrease in TNF-α production, both protein and mRNA-wise (62). They revealed a pivotal role for CUL4B as a regulatory factor that orchestrates the loading of TNF-α mRNPs containing TTP onto P-bodies, a process that is disrupted upon CUL4B depletion, redirecting TNF-α transcripts towards mRNA decay pathways. This finding is consistent with other studies showing that CUL4B KO in macrophages reduces LPS-induced TNF-α production (61, 62).

Recent studies have delved into the intricate mechanisms underlying the role of CUL4B in promoting macrophage migration and adhesion, with a particular focus on its upregulation of ITGA9 (63). The formation of a heterodimer between ITGA9 and integrin β1 constitutes a crucial juncture that modulates an array of cellular processes, encompassing adhesion, motility, specialization, viability, and clonal expansion, among others (64, 65). In the context of diabetic kidney disease (DKD), the accumulation of macrophages within the kidney is a critical factor in accelerating disease progression. These macrophages secrete a variety of inflammatory cytokines, oxidative species, and profibrotic agents, which contribute to renal damage and fibrosis (66, 67). T CUL4B plays a pivotal role in modulating macrophage behavior in the diabetic kidney. Specifically, CUL4B promotes the upregulation of ITGA9, which enhances macrophage migration and adhesion (63). This process is crucial for understanding the regulatory networks that govern macrophage behavior in DKD, highlighting CUL4B as a potential therapeutic target. The influence of CUL4B on macrophage migration and adherence constitutes an intricate mechanism, which hinges upon the enzymatic function of the CRL4B complex. Specifically, the interaction between CRL4B and Class I histone deacetylases (HDACs), such as HDAC1 and HDAC2, has been identified as a key mechanism that inhibits the transcription of miR-194-5p.This inhibition, in effect, results in the upregulation of ITGA9, which subsequently promotes macrophage migration and adhesion (63).

From an academic standpoint, these discoveries offer intriguing prospects for advancing research endeavors focused on deciphering the intricate molecular pathways that govern the macrophage-associated CUL4B-miR-194-5p-ITGA9 nexus. Therapeutic approaches directed at this axis, involving the utilization of miR-194-5p analogs or the application of ITGA9-targeting antibodies, signify encouraging pathways for advancing the creation of innovative therapies aimed at DKD. By disrupting the regulatory cascade that promotes macrophage-mediated damage, these interventions may offer a means to delay the progression of DKD and improve patient outcomes.

## 4 The role of CUL4B in disease pathogenesis

### 4.1 CUL4B limits myeloid-derived suppressor cells to enhance anti-tumor effects

CUL4B exhibits a dual role in cancer biology, being highly expressed in numerous solid tumors and essential for maintaining tumor malignancy, thereby promoting oncogenesis. Alternatively, it regulates the tumor’s local milieu by suppressing the gathering and activation of MDSCs, thereby prompting an anti-tumor response.

The MDSCs, which are characterized by their Gr-1+CD11b+ immunophenotype in murine systems, represent a pivotal cellular subpopulation within the intricate tapestry of the tumor microenvironment (TME). Recent advancements in cancer immunology have shed light on the multifaceted function of MDSCs in driving tumor progression through their immunosuppressive abilities, augmenting tumor cell growth, motility, and penetration, and also their pivotal role in fostering angiogenesis (68–74). Notably, CUL4B, a pivotal constituent within the E3 ubiquitin ligase complex, has surfaced as a critical inhibitory factor, exerting a negative regulation on the function of MDSCs, spanning across a wide range of tumoral contexts (75). By preventing the accumulation and suppressive activities of MDSCs, the key E3 ubiquitin ligase component CUL4B disrupts the formation of a tumor-permissive niche, thereby eliciting profound antitumor effects.

**Figure 3:**
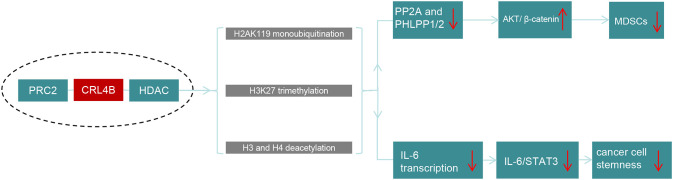
Schematic diagram of the anti-tumor mechanism of CRL4B/PRC2/HDAC complex. The CRL4B/PRC2/HDAC complex utilizes H2AK119 monoubiquitination, H3K27 trimethylation, and H3/H4 deacetylation to regulate gene expression. By downregulating PP2A and PHLPP1/2, it activates the AKT/GSK3β/β-catenin pathway, inhibiting the growth of MDSCs. Additionally, it reduces IL-6 transcription, suppressing the IL-6/STAT3 signaling, leading to a decrease in tumor stem cell properties and effectively exerting an anti-tumor effect.

CUL4B serves as a structural foundation for the CRL4B/PRC2/HDAC complex, which modulates gene expression through histone modifications (**Figure 3**). HDACs, key players in chromatin remodeling, deacetylate histones, leading to chromatin compaction and suppressed transcriptional activity (76). In cancer immunity, HDACs enhance immune signatures on neoplastic cells, boost immune effector function, and increase immune regulatory cytokines, thereby strengthening antitumor immunity (77). HDACs also transform MDSCs into dendritic cells and macrophages, reducing MDSC counts (78). T The CRL4B/PRC2/HDAC complex promotes H3/H4 deacetylation, H3K27 trimethylation, and H2AK119 monoubiquitination, disrupting the AKT/GSK3β/β-catenin signaling cascade by inhibiting AKT-specific phosphatases (PP2A and PHLPP1/2). This results in decreased MDSC accumulation and a less tumor-favorable microenvironment, highlighting CUL4B’s role in suppressing MDSC function and tumor growth The CRL4B/PRC2/HDAC complex promotes H3/H4 deacetylation, H3K27 trimethylation, and H2AK119 monoubiquitination, disrupting the AKT/GSK3β/β-catenin signaling cascade by inhibiting AKT-specific phosphatases (PP2A and PHLPP1/2). This results in decreased MDSC accumulation and a less tumor-favorable microenvironment, highlighting CUL4B’s role in suppressing MDSC function and tumor growth (75) (**Figure 3**).

In myeloid-specific CUL4B KO knockout models, augmented tumor growth and metastasis correlate with elevated IL-6 secretion by MDSCs. In myeloid cells, the CRL4B/PRC2/HDAC complex inhibits IL-6 transcription by promoting deacetylation of H3 and H4, H3K27 trimethylation, and H2AK119 monoubiquitination. By disrupting the IL-6/STAT3 interplay, this process further hinders the signaling pathway, thereby mitigating the stemness properties of tumor cells (**Figure 3**) (79). This further underscores the tumor-suppressing function of CUL4B in the context of MDSC-mediated tumor progression.

In an intriguing twist, the absence of CUL4B in intestinal epithelium accelerates the development of ApcMin/+ adenomas (80). This phenomenon is attributed to the collaborative action of CUL4B and PRC2, which bind to the Csf3 promoter and regulate histone modifications, including H2AK119 monoubiquitination and H3K27 trimethylation, to actively repress G-CSF transcription. In CUL4B-deficient intestinal epithelium, G-CSF, a potent recruiter of MDSCs, is upregulated, fostering a tumor-prone environment through enhanced MDSC recruitment and promotion of ApcMin/+ adenoma formation. However, neutralizing MDSC function restores the tumor-prone microenvironment, diminishing adenoma formation, thereby underscoring the pivotal role of MDSCs in modulating the consequences of CUL4B deficiency. The notable differences encountered in comparing *in vitro* and *in vivo* experimental settings, where CUL4B knockout in adenoma-derived organoids led to reduced organoid formation and size, contrast sharply with the *in vivo* effects, suggest a pivotal role for the TME matrix in modulating these outcomes. The introduction of MDSCs into the organoid culture system rescued the inconsistent phenotype, reversing Il6/Cox2 expression, and providing novel insights into the intricate interplay between CUL4B, MDSCs, and the TME in the context of ApcMin/+ adenoma development. These discoveries carry significant ramifications for the advancement of precision therapies focused on disrupting CUL4B-orchestrated pathways in colon and rectal cancer (CRC), notably within the context of Wnt signaling hyperactivity (80).

Considering the environment of KRAS-promoted lung adenocarcinoma, CUL4B exerts its tumor-suppressing capabilities via intricate epigenetic processes that encompass coordinated interplay with HDACs, ultimately inhibiting the transcription of C-X-C motif chemokine ligand 2 (CXCL2) (81). This focus on HDACs is justified as the current research on KRAS-promoted lung adenocarcinoma primarily highlights the role of HDACs in the epigenetic regulation of CXCL2, while the involvement of PRC2 in this context is less documented.CXCL2, a pivotal member of the CXC chemokine subfamily, orchestrates the recruitment of MDSCs and fosters angiogenesis via its engagement with the C-X-C motif chemokine receptor 2 (CXCR2) receptor (82, 83). The CRL4B-HDAC complex precisely orchestrates histone modifications, specifically histone deacetylation and H2AK119 monoubiquitination, thereby suppressing the transcriptional output of the Cxcl2 gene. In CUL4B-deficient settings within KRAS-mutated lung cancer cells, a derepression of CXCL2 expression ensues, which, through the CXCL2-CXCR2 signaling axis, augments MDSC recruitment. This, in turn, leads to a diminution of CD8+ and CD4+ T cells within the TME, ultimately accelerating tumor progression. To reinforce this concept, the utilization of the CXCR2 inhibitor SB265610 significantly mitigated the expedited tumor expansion and the accumulation of MDSCs, which were both induced by the knockdown of CUL4B. Furthermore, gemcitabine, a renowned antitumor agent, has exhibited its proficiency in specifically reducing the presence of MDSCs within tumor-harboring hosts, subsequently fortifying the immune system’s resistance against tumor growth (84). The strategic deployment of Gr-1-targeting neutralizing antibodies or the potent antitumor agent gemcitabine effectively contained the overall MDSC population, countered the decrease in the infiltration of both CD4+ and CD8+ T cells, and alleviated the phenotype of expedited tumor growth evident in LLC cells with CUL4B knockdown. These findings underscore the pivotal role of CUL4B in mediating tumor suppression through its regulation of CXCL2 and the subsequent modulation of MDSC dynamics.

Contrasting with the majority of previous studies that focused on the oncogenic roles of CUL4B, recent scientific advancements have unveiled a suppressive function of CUL4B under specific conditions. This suppressive function is achieved through its mediation in inhibiting the activation and accumulation of MDSCs. This discovery provides a novel and rigorous academic perspective for the intensive exploration of tumor initiation and progression mechanisms. Additionally, it opens up new avenues for the strategic development of TME modulation and cancer immunotherapy.

### 4.2 CUL4B in immune-related cancers and inflammation

CUL4B plays a significant role in various immune-related cancers and inflammatory conditions. In pleural mesothelioma, CUL4B overexpression is associated with worse patient outcomes, and its downregulation reduces tumor cell proliferation and increases cell death by regulating TGF-β1 expression (85). In human osteosarcoma cells, the activation of the TNF-α/NF-κB axis enhances CRL4BDCAF11 E3 ligase activity, leading to the degradation of the CDK inhibitor p21Cip1 and regulating cell cycle progression (86). In colitis-associated cancer (CAC), inflammation and DNA methylation-dependent down-regulation of miR-34b-5p mediate c-MYC expression and CRL4DCAF4 E3 ligase activity, promoting tumor development (87). Additionally, experimental autoimmune encephalomyelitis (EAE) serves as a model for multiple sclerosis (MS), highlighting the complex interaction between immunopathological and neuropathological mechanisms, and the role of CUL4B in these processes (88).

### 4.3 CUL4B in T cell regulation and autoimmune diseases

CUL4B plays a significant role in T cell regulation and autoimmune diseases. DDB1 is essential for the expansion of CD4 helper T cells by regulating cell cycle progression and cell death, highlighting its role in maintaining the balance of the immune system (4). In rheumatoid arthritis (RA), circ_0011058 alleviates pathology through the circ_0011058/miR-335-5p/CUL4B signal axis, suggesting a potential therapeutic target for RA (89). Additionally, CUL4B promotes the pathology of adjuvant-induced arthritis in rats through the canonical Wnt signaling, further emphasizing its role in autoimmune diseases (90).

### 4.4 CUL4B in metabolic diseases

CUL4B plays a significant role in metabolic diseases, particularly in the regulation of adipose tissue and insulin sensitivity (91). In adipocytes, the lack of CUL4B promotes PPARγ-mediated adipose tissue expansion and insulin sensitivity. CUL4B-RING E3 ligase (CRL4B) negatively regulates PPARγ by promoting its polyubiquitination and proteasomal degradation. Depletion of CUL4B leads to upregulation of PPARγ-regulated genes and facilitates adipogenesis. Adipocyte-specific Cul4b knockout (AKO) mice fed a high-fat diet exhibit increased body fat accumulation mediated by increased adipogenesis. However, AKO mice show improved metabolic phenotypes, including increased insulin sensitivity and glucose tolerance. Correspondingly, there is a decreased inflammatory response in adipose tissues of AKO mice. Genetic inhibition of CUL4B thus appears to phenocopy the beneficial effects of PPARγ agonists. Collectively, this study establishes a critical role of CRL4B in the regulation of PPARγ stability and insulin sensitivity and suggests that CUL4B could be a potential therapeutic target for combating obesity and metabolic syndromes (91).

## 5 Conclusion

CUL4B has attracted considerable interest within the realm of oncology, primarily due to its pervasive overexpression across numerous human malignancies, which has been persistently linked to tumor advancement and aggressiveness (3, 92–96). Paradoxically, in the context of immune cells, CUL4B exhibits an opposing function, as evidenced by the acceleration of oncogenic transformation observed in hematopoietic or myeloid cells specifically deficient in CUL4B (75, 79). Mechanistically, CUL4B wields its inhibitory influence on tumor progression through the manipulation of the immune microenvironment, thereby exerting tumor-restraining effects. Specifically, it impedes the accrual and activation of MDSCs, thereby mitigating their immunosuppressive effects on T cells and preserving IL-6 levels. Subsequently, it diminishes the fostering of a microenvironment that is permissive for tumor growth and metastasis, thereby hindering its progression.

The CRL4B complex, of which CUL4B is a central component, has two primary functions: the ubiquitination of substrate proteins leading to their degradation, and the monoubiquitination of H2AK119 leading to transcriptional repression. While the role of CRL4B in transcriptional repression has been well-studied (3, 92–96), the discussion of CRL4B-mediated protein degradation is relatively limited. This may not be due to the lesser importance of protein degradation in immune cell regulation, but rather an unintentional oversight in the research focus. Given the critical role of protein degradation in maintaining cellular homeostasis and regulating immune responses, it is necessary to conduct a detailed discussion on this topic. Future research should aim to fill this gap by exploring the specific substrates targeted by CRL4B for degradation and their impact on immune cell function (4, 6).

This review highlights the pivotal role of CUL4B in maintaining immune homeostasis and provides novel perspectives and insights into the understanding and development of treatments for immune-related disorders. By exploring the latest findings and discussing potential avenues for future research, we hope to contribute to the ongoing efforts to unravel the complex mechanisms underlying immune regulation and disease pathogenesis.
